# Finishing and Polishing of Composite Restoration: Assessment of Knowledge, Attitude and Practice Among Various Dental Professionals in India

**DOI:** 10.7759/cureus.20887

**Published:** 2022-01-03

**Authors:** Sankar Vishwanath, Sadasiva Kadandale, Senthil kumar Kumarappan, Anupama Ramachandran, Manu Unnikrishnan, Honap manjiri Nagesh

**Affiliations:** 1 Conservative Dentistry and Endodontics, Chettinad Dental College and Research Institute, Chennai, IND

**Keywords:** composite restoration, finishing and polishing, oxygen inhibition layer, polishing paste, surface sealants, indian dentists

## Abstract

Aim

The key to the success of a composite restoration lies in the important final step of finishing and polishing. This survey aims to assess the knowledge, attitude, and practice about finishing and polishing in composite restoration among various dental professionals in India.

Materials and methods

A cross-sectional questionnaire survey was conducted among various dental professionals in India. The participation of dentists was voluntary. A total of 350 responses were received, and the data were converted to Microsoft Excel 2019 program and descriptive statistics were analyzed using SPSS Version 21.0 software.

Results

Almost 99% of respondents know the importance of finishing and polishing procedures of composite restoration. Overall, 71% of respondents felt it is necessary to remove the surface layer to improve the surface characteristics of the composite restoration, 59.8% of professionals follow the sequential order of abrasives for finishing and polishing of composites, 74.2% use interproximal strips to finish interproximal areas of restoration, and 55.8% of professionals use polishing paste for the final polishing of composite restoration. Only 44.2% are aware of liquid polish/composite glaze, among which 12.4% of practitioners use liquid polish often.

Conclusion

Most of the respondents were aware of the benefits of proper finishing and polishing of composite restorations. However, still they need to follow proper sequential series of burs, abrasive points, disks, strips, and polishing pastes. The usage of surface sealants should be emphasized for enhanced results.

## Introduction

Aesthetic restorations are an inseparable part of modern-day dental practice [1]. Restoration with composite material, like elsewhere, is the most popular and commonly used aesthetic restorative material in India. In recent years, the usage of the composite as a choice of restorative material has increased incredibly because of its versatile combination of aesthetics, affordability, and conservation. Thus, composite restorations are preferred in both anterior and posterior teeth [2]. The overall survival rate of composite restorations is 75.6%after 10 years [3]. The most common reason that necessitates replacement of composite restoration is color change, fracture, and marginal degradation leading to secondary caries [4]. Composite has a good survival rate and longevity if proper compliance with the treatment protocol is followed [5]. The key to aesthetics and bio-integration of composite restorations lies in the final step of finishing and polishing, which is an important step of the clinical restorative procedure. Therefore, to achieve a successful restoration, it is important to maintain a smooth surface [6]. Ideal finishing and polishing of restorations improves aesthetics and maintains the oral health of a patient [7,8]. A smooth surface is needed to provide comfort to the patient and reduce irritations to cheeks, tongue, and lips. Patient consciousness of restorations increases as the tip of the tongue can detect alteration in surface roughness of 0.3 μm [9]. A rough surface serves as a site for bacterial adhesion, accumulates plaque, causes irritation to the gingiva, and results in periodontal diseases [10]. This increases surface staining and affects esthetics [7,11]. Increased microleakage can cause staining at restoration margins, sensitivity, and secondary caries formation [12]. It also causes wear of opposing and adjacent teeth [2]. The dental practitioner's knowledge and attitude toward oral health provides the framework to deliver an effective evidence-based treatment. This survey aims to assess the knowledge, attitude, and practice about finishing and polishing of composite restoration among various dental professionals in India.

## Materials and methods

Study design

A cross-sectional study was designed based on the STROBE (STrengthening the Reporting of OBservational studies in Epidemiology) guidelines.

Inclusion and exclusion criteria

General dental practitioners, postgraduate students, and academicians were included in this survey. Students pursuing under graduation were excluded.

Data collection

After obtaining ethical approval from the Chettinad Academy of Research and Education Institutional Human Ethics Committee for Students Research (IHEC-I/0118 /21), a cross-sectional questionnaire survey was carried out among various dental professionals in India. A sample size of 350 was estimated using the G*Power software. The web link of the questionnaire in the form of Google Forms was prepared in English and circulated through online medium after validation. The electronic questionnaire contained details about the purpose of the study and an informed consent before entering to the question section. The participation of dentists was voluntary, and the data collection maintained confidentially. Demographic details such as age, years of clinical experience, and type of practice were collected before the questionnaire section. The structured questionnaire had four knowledge-based questions, three attitude questions, and 14 practice questions in a mixed manner with a sequential flow. All the questions in the study were framed suitably in multiple-choice pattern with column provided for writing their answers wherever applicable and appropriate. One question was open-ended to know what composite finishing and polishing system or kit the practitioner uses. As per the estimated sample size, the responses from 350 dental professionals were obtained within a time period of 15 days.

Statistical analysis

The response to the questionnaire was received in the form of spreadsheet. The data were converted to Microsoft Excel 2019 program, and descriptive statistics such as percentages were analyzed using SPSS Version 21.0 software (IBM Corp., Armonk, NY).

## Results

Of the total survey respondents, 45.3% of participants were general practitioners, 44.2% were postgraduate students, 6% were academicians and practitioners, and 4.6% were specialty dental practitioners. Among the survey participants, 86% had less than five years of clinical experience, 8% of participants had 6 to 10 years of clinical experience, and 5% of participants had more than 10 years of clinical experience. Overall, 99.1% of respondents considered finishing and polishing is an essential step in enhancing the clinical longevity of composite restoration.

**Figure 1:**
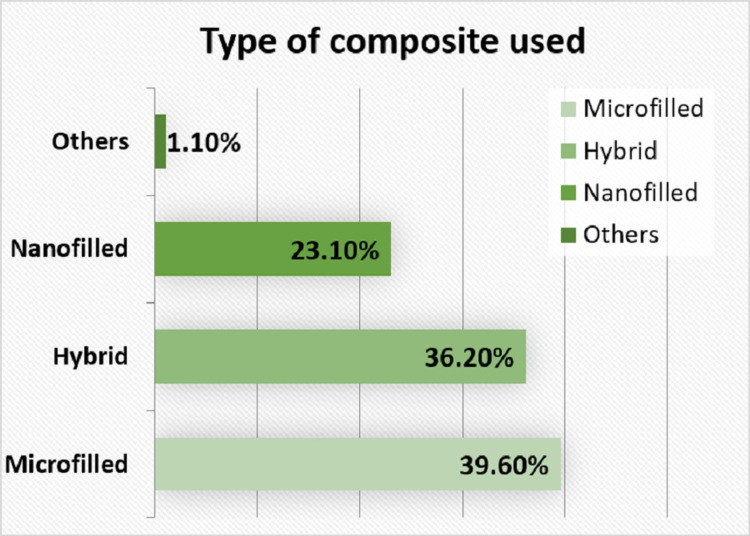
Type of composite used by practitioners

For a question on which material according to respondent gives superior surface properties, 55% (193) suggested nano-filled composite, 24.2% (85) suggested hybrid composite, and 20.2% (71) suggested microfilled variety. Figure 1 represents the type of composite used by the respondents.

**Figure 2:**
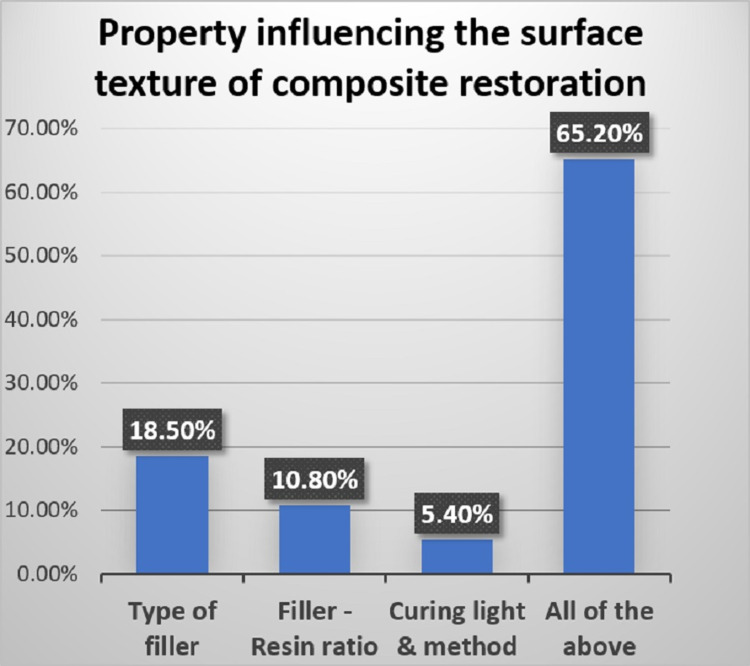
Property influencing the surface quality of composite restoration

**Figure 3:**
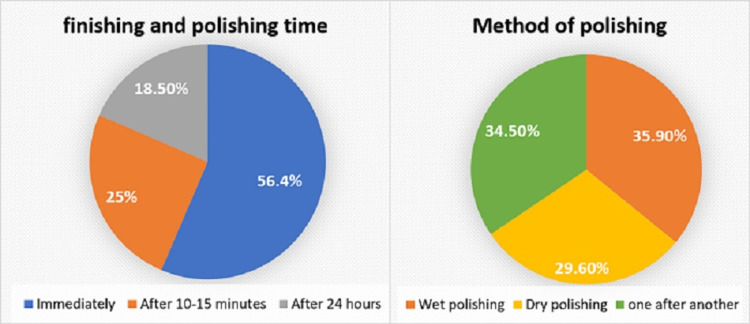
Timing and method of finishing and polishing

Response to a question on properties that affect the surface texture of a composite restoration can be seen in Figure 2. For the question on the ideal time to finish and polish composite restoration and on preferred method of finishing and polishing, the responses are represented in Figure 3.

 

As shown in Table 1, it is evident that 72.9% (256) of respondents know the sequential order of abrasives and 59.8% (210) are following this sequential order during finishing and polishing procedure. Results reveal that 68.1% (239) of respondents finish and polish interproximally (Table 1); 74.2% (190) use interproximal strips to finish interproximal areas of restoration, and the rest of them use fine diamond yellow bur, 16% (41), and abrasive disk, 9.8% (25). Also, 30.8% (108) of respondents use an occlusal brush for the occlusal surface of posterior composite restoration; 55.8% (196) use polishing pastes (Table 1); 69.7% (147) use a rubber polishing cup to apply polishing paste followed by wheels, disc and spirals 16% (34), and brush 13.7% (29); and 44.2% (155) are aware of liquid polish/composite glaze (Table 1) among which only 12.4% (28) of practitioners use liquid polish often, 31.9% (72) use occasionally, and more than 55.8% (126) professionals never use it. Table 2 depicts the response to practice-based questions on instruments used for finishing of composite restorations.

**Table 1:** Response to attitude, knowledge, and practice-based questions % (n): percentage of response (number of responses)

Respondents opinion for attitude-based questions			
Question	Responses % (n)		
	Yes	No	Not sure
Do you think finishing and polishing procedure is an essential step in enhancing clinical longevity of composite restoration?	99.1% (348)		0.9% (3)
Removal of the surface layer is necessary to improve the surface characteristics of a composite restoration	70.7% (248)	6.6% (23)	22.8% (80)
Response to knowledge-based questions			
Question	Responses % (n)		
	Yes	No	
Do you know about the oxygen-inhibited layer formed over the surface of cured composite restoration?	69.8% (245)	30.2% (106)	
Do you know the various grit sizes and color-coding of abrasives?	72.9% (256)	27.1% (95)	
Do you know about liquid polish/composite glaze used as a final application for composite restoration?			
Response to practice-based questions			
Question	Responses	% (n)	
	Yes	No	
Are you following the sequential order of abrasives in the finishing and polishing of composites?	59.8% (210)	40.2% (141)	
Do you slightly overfill the cavity margins to compensate for removal during finishing and polishing?	78.6% (276)	21.4% (75)	
Do you finish and polish interproximally?	68.1% (239)	31.9% (112)	
Do you use an occlusal brush for the occlusal surface of posterior composite restoration?	30.8% (108)	69.2% (243)	
Do you use polishing paste for final polishing?	55.8% (196)	44.2% (155)	

**Table 2:** Response to three practice-based questions % (n): percentage of response (number of responses)

Question	Responses % (n)			
Bulk reduction and/or contouring	Coarse diamond bur (blue/green-coded)	12 fluted carbide burs	Coarse abrasive disc	Other: fine yellow diamond
	59.80% (210)	22.20% (78)	16% (56)	2% (7)
Finishing of facial/lingual surfaces of anterior composite restoration	Fine diamond bur (yellow-coded)	16-30 fluted carbide bur	Abrasive wheels and points	Abrasive coated disc
	59.50% (209)	22.20% (78)	13.70% (48)	4.6% (16)
Finishing of occlusal surface of posterior composite restoration	Fine diamond bur (yellow-coded)	16-30 fluted carbide bur	Abrasive wheels & points	Abrasive coated disc
	52.40% (184)	12.80% (45)	28.80% (101)	6% (21)

For an open-ended question on what finishing and polishing system is used by the practitioner, 147 responses were obtained, out of which a maximum of 65 respondents mentioned the SHOFU polishing kit (SHOFU, San Marcos, CA), followed by Sof-Lex™ (10; 3M, St. Paul, MN), and a few mentioned Super-Snap® (SHOFU).

## Discussion

The present study is the first survey to assess the knowledge, attitude, and practices regarding the finishing and polishing procedure in composite restorations among dental professionals in India.

Almost 99% of respondents know the importance of finishing and polishing steps in composite resin restoration. Studies have shown that the operator age and clinical experience did not influence the quality of finishing and polishing procedures [8,13]. In the present study, data were obtained from dental practitioners from different age groups and various years of clinical experience. Most of the respondents were within the age group of 24 to 30 years (292 out of 350), and 86% (295) of participants had a clinical experience of five years and lesser.

Oxygen-inhibited layer (OIL) is a soft, sticky superficial layer always present when the composite is polymerized in contact with air [14,15]. This layer is comprised of unpolymerized or poorly polymerized resin monomers that would be more susceptible to staining when it comes in contact with food and beverages. Therefore, removal of OIL by finishing and polishing procedures is mandatory to provide an esthetically durable surface that is resistant to staining [16]. Overall, 70% of respondents knew about OIL and 71% felt that it is necessary to remove the surface layer (Table 1). Curing against Matrix bands and Mylar strips would block atmospheric oxygen from the surface of the restoration [17]. However, the matrix strip alone is not enough to provide the proper anatomic contours of the composite restoration. Thus, in most cases, removal of the superficial layer becomes a necessary step.

As felt by 65.20% of participants (Figure 2), several studies show that the surface quality of composite is influenced by various intrinsic factors such as the type of material, type of filler, distribution of filler, the polymerization procedure, and extrinsic factors such as finishing polishing systems and technique applied [8,18,19].

Regarding finishing and polishing time (Figure 3), 56.4% of respondents finish and polish immediately, 25% of respondents finish after 10-15 minutes, and 18.5% of respondents finish after 24 hours. The time elapsed between the placement of restoration and finishing is always a debate. Immediately after curing, the material is not completely mature [12,20]. Thus, the heat generated would result in the flow of the material and deform the composite, thereby compromising the initial marginal sealing [20]. Earlier studies stated that improved marginal seal is obtained by polishing after 24 hours. But delayed polishing could damage the seal obtained by hygroscopic expansion of the composites and increase the microleakage [12,21]. Most dentists choose to do the finishing and polishing step at the same sitting immediately after restoration placement, which is more convenient and acceptable for the patient [22]. Therefore, finishing should be delayed as long as is practical to minimize the adverse effects. Finishing after 10-15 minutes is advised. Approximately 75% of the polymerization of photo-polymerized composite resins takes place during the first 10 minutes, but this curing reaction may continue for 24 hours [23].

Many prefer to finish and polish without any coolant since it allows better visualization of the restoration margin (Figure 3). The disadvantages of the dry method are structural and chemical changes on the surface of restorations [20]. The heat produced can create a lot of cracks and excessive roughness on the surface of the restoration. Moreover, it affects marginal sealing and increases microleakage [21,24]. Clinicians should finish the restoration in an environment in which margins are discernible and where minimal heat is generated. Therefore, it is recommended to finish and polish the composite under water coolant to reduce the detrimental effects of dry finishing and polishing [20].

The finishing-polishing procedure involves a sequential use of instruments with a progressive decrease in abrasiveness to achieve a highly smooth surface [18]. It is essential for the practitioners to know and follow the proper finishing sequence in the multistep process [8]. In this study, 59.8% (210) respondents are following a sequential method. Many one-step polishing systems for composite have been introduced in the market and are efficacious, such as multiple-step polishing systems. This single-instrument system conserves the time of clinicians [2].

In accordance with results, diamond abrasive burs are suitable for bulk reduction and contouring due to their highly efficient cutting action on composite surfaces. Due to low efficiency in cutting, carbide finishing burs are apt for smooth finishing of the composite surfaces [25]. From Table 2, it is evident that yellow-coded diamond burs with fine-grit abrasives (20-40 μm) are preferred by most of the respondents for finishing of facial, lingual/palatal surfaces of anterior composite restorations, 59.5% (209), and for finishing the occlusal surface of posterior composite restoration, 52.40% (184). Thus, it is suggested to use egg-shaped or barrel-shaped yellow-coded finishing burs with fine abrasives (20-40 μm) to selectively sculpt away excess without significantly affecting marginal integrity [26]. For instrumentation of anatomically structured surfaces such as the occlusal surface of the posterior teeth, polishing brushes are used [26]. A new polishing brush with ‘‘bristles’’ incorporating silicon carbide particles is available in pointed and cup shape. They produced good surface smoothness and a surface luster by gaining access to the pits, fissures, and interproximal areas of composite restorations that remain unreached with other instruments [2].

Of the respondents, 74.20% (190) use interproximal strips. Proper contouring by matricing techniques reduces postoperative finishing and polishing of proximal surfaces. The use of flexible disks is more challenging in the interproximal region because of the anatomic shape, difficult access, and visibility. Fine-diamond abrasive polishing strips are ideal for additional polishing of convex or flat proximal surfaces and embrasure areas [26,27].

According to Jefferies [2], applying fine abrasive paste as the final step of polishing composite restoration after using sequential abrasive coated discs produces a highly smooth and glossy surface. The composition of the paste, the design of the application device, and the manner of application of paste are important in the polishing procedure [2]. For application, 69.7% (147) use a rubber polishing cup followed by wheels, disc, and spirals, 16% (34), and brush, 13.7% (29). A flexible rubber polishing cup is often used for applying the polishing paste, but studies suggest the use of specialized shapes of abrasives such as soft foam or felt applicators can significantly enhance the effect of polishing paste [2,28].

In the study, only 44.2% (155) are aware of liquid polish/composite glaze, among which only 12.4% (28) of practitioners use liquid polish often. Microcracks resulting from trauma due to finishing procedures particularly at the cavosurface margins can propagate over time [29]. Liquid polishers/surface sealants are low-viscosity resins with little or no filler that provide a gloss over composite restorations improving the final appearance of the restoration. Sealants fill irregularities and reduce microleakage at composite margins [30]. Thus, sealant application may prevent surface wear, thereby improving the longevity of composite restorations [29]. If needed, sealant can be reapplied biannually [27]. This is a simple method to improve surface quality but an additional step for the dentist to complete the procedure.

The present study, however, has a few limitations that include non-uniformity in participation among the respondents. Also, the number of individuals who responded is quantifiably low to arrive at a definitive conclusion.

## Conclusions

Since composite is the most commonly used restorative material in clinical practice, dental professionals should know the importance and sequential use of finishing and polishing. It is necessary that dentists must be equipped with knowledge regarding newer advancements. There is a need for continuing dental education programs and workshops to upskill practicing dentists with proper techniques and implementing that in private practice setup. Importance of using polishing paste and liquid polish should be emphasized in the undergraduate curriculum.

## Appendices

Questionnaire

1)    Do you think finishing and polishing procedure is an essential step in enhancing clinical longevity of composite restoration?

a.     Yes

b.    No

c.     Not sure

2)    Do you know about oxygen-inhibited layer formed over surface of cured composite restoration?

a.     Yes

b.    No

3)    Removal of the surface layer is necessary to improve the surface characteristics of a composite restoration

a.     Yes

b.    No

c.     Not sure

4)    According to you, which composite material gives superior surface properties

a.     Microfilled

b.    Hybrid

c.     Nanofilled

d.    Others   mention_________

5)    what composite do you use?

a.     Microfilled

b.    Hybrid

c.     Nanofilled

d.    Others mention_________

6)    Which of the following properties influences the surface texture of composite restoration?

a.     Type of filler

b.    Filler - Resin ratio

c.     Curing light & method

d.    All of the above

7)    What will be your finishing and polishing time after a composite restoration?

a.     Immediately

b.    After 10-15 minutes

c.     After 24 hours

8)    What method do you prefer?

a.     Dry polishing

b.    Wet polishing

c.     One after another

9)    Do you know the various grit sizes and color coding of abrasives?

a.     Yes

b.    no

10)    Are you following the sequential order of abrasives in finishing and polishing of composites?

a.     Yes

b.    No

11)     Do you slightly overfill the cavity margins to compensate removal during finishing and polishing?

a.     Yes

b.    No

12)    What will you use for bulk reduction and/or contouring?

a.     Coarse diamond bur (blue/green coded)

b.    12 fluted carbide burs

c.     Coarse abrasive disc

d.    Others   mention_________

13)    What will you use to finish facial/ lingual surfaces of anterior composite restoration?

a.     Fine diamond bur (yellow coded)

b.    16-30 fluted carbide bur

c.     Abrasive wheels & points

d.    Abrasive coated disc

14)    What will you use to finish occlusal surface of posterior composite restoration?

a.     Fine diamond bur (yellow coded)

b.    16-30 fluted carbide bur

c.     Abrasive wheels & points

d.    Abrasive coated disc

15)   Do you finish & polish interproximally?

a.    Yes

b.    No

16)   If yes, what will you use?

a.    Fine diamond bur (yellow coded)

b.    Interproximal strips

c.    Abrasive disc

d.    Other ­­­­­­   mention_________

17)   Do you use an occlusal brush for occlusal surface of posterior composite restoration?

a.    Yes

b.    No

18)   Do you use polishing paste for final polishing?

a.    Yes

b.    No

19)  If yes, how do you apply it?

a.   Rubber polishing cup

b.   Wheels, discs, and spirals

c.    Brush

d.    Others mention_________

20)  Do you know about liquid polish / composite glaze used as final application for composite restoration?

a.     Yes

b.    no

21)   If yes, how frequently you use it?

a.     Often

b.    Occasionally

c.     Never use it

22)   Name the composite finishing and polishing system/kit you use _____________
